# Perioperative testosterone therapy linked to higher 5-year risk of periprosthetic joint infection without increased 90-day major complications or revision rates in total shoulder arthroplasty

**DOI:** 10.1177/17585732261437972

**Published:** 2026-04-07

**Authors:** Jad J Lawand, Connor Crutchfield, Alejandro M Holle, Olawale A Sogbein, Sameer R Khawaja, Umar Ghilzai, Brian W Hill, Adam Z Khan, John G Horneff, Joseph A Abboud

**Affiliations:** 1 Department of Orthopaedic Surgery and Rehabilitation, 441907 University of Texas Medical Branch, Galveston, TX, USA; 2 12313 Department of Orthopaedic Surgery, Sidney Kimmel Medical College at Thomas Jefferson University, Philadelphia, PA, USA; 3 503668 Department of Orthopaedic Surgery, Mayo Clinic Alix School of Medicine, Phoenix, AZ, USA; 4 Division of Shoulder and Elbow Surgery, 387400 Rothman Orthopaedic Institute, Philadelphia, PA, USA; 5 Josephbart Department of Orthopaedic Surgery, 3989 Baylor College of Medicine, Houston, Texas, USA; 6 Department of Orthopaedic Surgery, 6559 Palm Beach Orthopaedic Institute, Palm Beach Gardens, FL, USA; 7 Department of Orthopaedic Surgery, 160623 Southern California Permanente Medical Group, Panorama, CA, USA; 8 Department of Orthopaedic Surgery, 387400 Hospital of the University of Pennsylvania, University of Pennsylvania, Philadelphia, PA, USA

**Keywords:** Shoulder arthroplasty, revision shoulder arthroplasty, testosterone, complications, periprosthetic joint infection, shoulder replacement, reoperation

## Abstract

**Introduction:**

Preoperative testosterone exposure has been associated with various health outcomes, but its impact on postoperative complications in patients undergoing total shoulder arthroplasty (TSA) has not been fully explored. This study aims to evaluate the association between preoperative testosterone exposure and postoperative medical and surgical complications in patients undergoing TSA.

**Methods:**

A retrospective cohort study was conducted using the PearlDiver database. Patients who underwent TSA and used preoperative testosterone within 3 months of the index procedure were identified and matched 1:4 to controls without testosterone use using propensity score matching. Matching was performed based on age, gender, comorbidities, Charlson comorbidity index, obesity, tobacco use, and hypogonadism to balance the groups. Postoperative complications were assessed at 90 days and five years. Statistical comparisons were made using odds ratios (ORs) with 95% confidence intervals (CIs), and a *p*-value of <0.05 was considered statistically significant.

**Results:**

The matched cohort included 677 testosterone users and 2554 controls. There were no statistically significant differences between the testosterone and control groups in 90-day complications such as myocardial infarction (OR 2.52, *P* = 0.31), pneumonia (OR 0.86, *P* = 0.70), and deep vein thrombosis (no cases in the testosterone group). Over five years, the testosterone group had a significantly higher risk of periprosthetic joint infection (4.1% vs. 2.4%, OR 1.76, 95% CI 1.10–2.75, *P* = 0.015), but no differences in revision rates, dislocation, aseptic loosening, or stiffness were observed.

**Conclusion:**

Preoperative testosterone exposure was associated with a higher risk of periprosthetic joint infection following TSA, but no significant differences were found in major medical complications or revision rates.

## Introduction

The incidence of total shoulder arthroplasty (TSA) has been increased more than 3-folds over the past two decades, with annual procedure volumes expected to exceed 140,000 cases by 2025.^1–6^ Most commonly indicated in cases of rotator cuff arthropathy and severe osteoarthritis, TSA is performed when non-operative treatment fails to relieve pain or improve function. Notably, patients who undergo TSA commonly do so in the seventh decade of life, and with the average age of the United States continuing to rise, the number of arthroplasty procedures performed annually will likely grow with it.^7^

Interestingly, the prevalence of testosterone replacement therapy (TRT) in patients undergoing TSA has also been increasing.^8,9^ While TRT has various benefits for individuals with declining levels of endogenous testosterone,^8,10,11^ there is also evidence it may produce deleterious health effects. Prior investigations of TRT demonstrated adverse events across multiple organ systems, including cardiovascular, respiratory, integumentary, and musculoskeletal complications.^12–15^ Notably, in one randomized trial, men receiving TRT were twice as likely as those receiving placebo to require medical evaluation for an adverse event.^12^ Together, such findings suggest that testosterone supplementation is not without risk and that its increasing use in TSA patients requires further investigation, particularly with regard to infection.

Periprosthetic joint infection (PJI) after TSA is a rare though still devastating complication of surgery, occurring in approximately 0.008%–3.9% of cases.^9,16–22^ While numerous factors predictive of PJI have been reported in the literature, it has been well-documented that male sex is a strong risk factor.^16,19,23–27^ Leading theories for this implicate the increased level of serum testosterone in males, which increases sebum production and the burden of *Cutibacterium acnes* by providing its preferred lipid-rich anaerobic environment for proliferation.^27,28^ Findings by Falconer et al.^25^ have also supported this, demonstrating that males undergoing primary TSA were on average 66 times more likely to have an intraoperatively collected tissue culture positive for *C. acnes* contamination (*P* < 0.001). In addition, Schiffman et al.^27^ demonstrated using multivariate analysis that serum testosterone was an independent predictor of high skin *Cutibacterium* loads (*P* < 0.001), further reinforcing a link between testosterone levels, *C. acnes,* and PJI.^29^

Exogenous testosterone use has also been associated with PJI after primary TSA. In a study by Su et al.^9^ examining the use of TRT on TSA infection rates, male patients who used testosterone within 6 months prior to surgery had a higher rate of infection at 2 years postop than the controls (3.4% vs. 2.4%, *P* = 0.042). The prior study by Schiffman et al.^27^ also found that patients taking supplemental testosterone had higher free testosterone levels and tended to have higher skin *Cutibacterium* loads. However, TRT's impact on postoperative outcomes in patients undergoing primary TSA is still not well understood. Though prior studies have begun to outline an association between TRT and infection risk,^9,27,29^ the two studies that have investigated a direct relationship were insufficiently powered due to short-term outcomes^9^ or few subjects,^29^ and neither used cohort matching. This study aims to evaluate the association between preoperative testosterone exposure and postoperative medical and surgical complications in patients undergoing primary TSA. We hypothesize that testosterone supplementation will increase rates of infection and prosthetic-related complications but will not significantly affect long-term rates of medical complications.

## Methods

### Study design and database

A retrospective cohort study was conducted using the Mariner dataset from the PearlDiver Patient Records Database (PearlDiver, Colorado Springs, CO, USA), which contains de-identified claims data for over 151 million patients between January 2010 and April 2023. The dataset allows for comprehensive follow-up using unique patient identifier codes, ensuring continuity of patient records regardless of changes in insurance status. Because the data was de-identified, this study was exempt from Institutional Review Board (IRB) approval.

### Patient population

Patients who underwent primary TSA were identified using specific Current Procedural Terminology and International Classification of Diseases (ICD) procedure codes (Supplemental Table 1). Inclusion criteria required patients to have at least five years of postoperative follow-up, confirmed by reviewing patient records for continuous insurance coverage. Patients were excluded if they were previously diagnosed with a proximal humerus fracture or malignancy. Additionally, patients with less than five years of follow-up were excluded, as were those who underwent contralateral TSA within five years of the index procedure, to maintain the focus on outcomes from single shoulder surgery. The study population was divided into two groups: those who received preoperative TRT within three months of the surgery, and a control group of patients who did not receive TRT. Perioperative TRT exposure was identified using National Drug Codes for testosterone prescriptions and ICD-9/10 diagnosis codes for testosterone administration during outpatient encounters. A patient was considered exposed if any TRT prescription fill or administration record occurred within 3 months before surgery.

### Matching procedure

**Figure 1.**
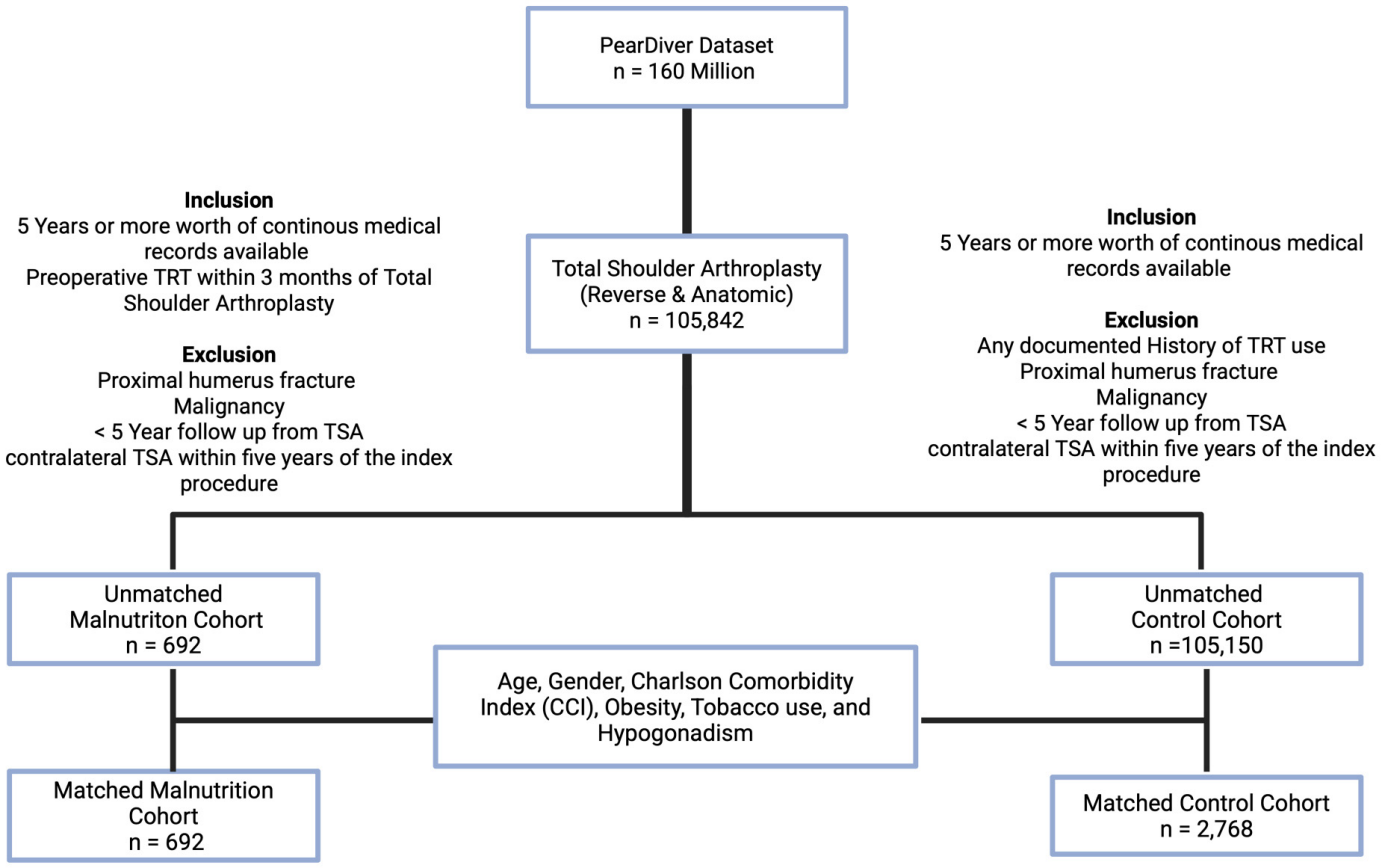
Flowchart demonstrating inclusion, exclusion, and matching processes.

To reduce bias and ensure comparability between the TRT and control groups, propensity score matching was performed. Matching variables were selected based on prior evidence linking them to postoperative complications after TSA (age, sex, Charlson Comorbidity Index (CCI), obesity, tobacco use, hypogonadism). Variables such as diabetes, hypertension, and hyperlipidemia were considered but were not included because they are encompassed within CCI. We elected to avoid overfitting the matching model, which can compromise match quality and produce cohorts. The variables used for matching included age, gender, CCI, obesity, tobacco use, and hypogonadism. This matching process resulted in two well-balanced groups, with no statistically significant differences in baseline demographics or clinical variables. Because 91% of TRT users continued therapy postoperatively, this study represents an evaluation of perioperative TRT exposure, not solely preoperative use. Matching variables were selected a priori based on prior evidence linking them to postoperative complications following TSA, including age, sex, CCI, obesity, tobacco use, and hypogonadism. Additional comorbidities such as diabetes, hypertension, and hyperlipidemia were considered but were not included as separate matching variables because they are incorporated within the CCI. We intentionally limited the number of matching variables to avoid overfitting the propensity model, which can reduce match quality and generalizability without improving confounder control (Figure 1).

### Statistical analysis

Demographics and comorbidities were compared between groups using *t*-tests for continuous variables and Chi-square tests for categorical variables. Postoperative outcomes were evaluated over a minimum follow-up period of five years. The analysis focused on both medical and surgical complications following TSA. Statistical comparisons between the TRT and control groups were conducted using odds ratios (ORs) with 95% confidence intervals (CIs). A *p*-value of less than 0.05 was considered statistically significant. All analyses were performed using *R* software (R Foundation for Statistical Computing, Vienna, Austria) provided within the PearlDiver database. As this was a retrospective claims-based study using all eligible patients meeting inclusion criteria, an a priori sample size target was not specified as it reflects the largest cohort available for analysis.

### Outcome measures

The primary outcomes of interest included five-year postoperative complications, such as all-cause revision, PJI, dislocation, aseptic loosening, periprosthetic fracture, and joint stiffness. Secondary outcomes included other major medical complications, including myocardial infarction, pneumonia, pulmonary embolism, deep vein thrombosis, urinary tract infection, acute kidney injury, blood transfusion, wound dehiscence, emergency department visits, and hospital readmission assessed during the follow-up period.

## Results

### Pre and post propensity matched cohorts

The initial unmatched cohort consisted of 692 testosterone users and 10,515 controls. After propensity score matching, the final matched cohort included 677 testosterone users and 2554 controls. In the unmatched cohort, testosterone users were younger (mean age 63.7 vs. 68.2 years, *P* < 0.001), more likely to be male (87.2% vs. 42.5%, *P* < 0.001), and had higher rates of obesity (49.0% vs. 44.3%, *P* = 0.015) and tobacco use (50.3% vs. 43.0%, *P* < 0.001) compared to controls. After matching, these differences were no longer significant, with comparable age (64.0 vs. 64.4 years, *P* = 0.21), gender distribution (87.0% male in testosterone group vs. 86.0% in controls, *P* = 0.53), obesity (48.6% vs. 48.1%, *P* = 0.85), and tobacco use (49.8% vs. 50.7%, *P* = 0.71) between the two groups, as depicted in Table 1. Among these patients, 631 (91%) continued TRT after TSA. Therefore, this study represents an evaluation of perioperative TRT exposure, not solely preoperative use.

**Table 1.** Demographic variables compared between matched and unmatched groups.

	Unmatched			Matched		
Variable	Testosterone (*n* = 692)	Controls (*n* = 10,515)	*P*-value	Testosterone (*N* = 677)	Controls (*n* = 2554)	*P*-value
Age (SD)	63.7 (8.3)	68.2 (7.7)	**<0**.**001**	64.0 (8.1)	64.4 (7.7)	0.21
Male	604 (87.2)	44680 (42.5)	**<0**.**001**	589 (87.0)	2196 (86.0)	0.53
Female	88 (12.7)	60470 (57.5)	**<0**.**001**	88 (13.0)	358 (14.0)	0.54
CCI (SD)	1.80 (2.25)	1.66 (2.10)	0.091	1.73 (2.1)	1.59 (1.9)	0.13
Obesity	339 (49.0)	46574 (44.3)	**0**.**015**	329 (48.6)	1228 (48.1)	0.85
Tobacco use	348 (50.3)	45223 (43.0)	**<0**.**001**	337 (49.8)	1294 (50.7)	0.71
Hypogonadism	509 (73.6)	4157 (4.0)	**<0**.**001**	494 (73.0)	1822 (71.3)	0.43

*Reported as *n* (%) unless otherwise specified. SD: standard deviation; CCI, Charlson Comorbidity Index. Bold indicates statistical significance (*P* < 0.05).

### 90-Day postoperative complications

In the matched cohort, there were no statistically significant differences in 90-day medical complications between testosterone users and controls as detailed in Table 2. The incidence of myocardial infarction was low in both groups (0.3% vs. 0.1%, OR 2.5, *P* = 0.31), and no cases of pulmonary embolism or deep vein thrombosis were observed in the testosterone group. Similarly, there were no significant differences in pneumonia (1.2% vs. 1.4%, OR 0.9, *P* = 0.70), urinary tract infection (1.9% vs. 1.3%, OR 1.5, *P* = 0.22), acute kidney injury (0.9% vs. 1.0%, OR 0.9, *P* = 0.83), emergency department visits (8.4% vs. 7.0%, OR 1.2, *P* = 0.22), or readmissions (1.9% vs. 2.9%, OR 0.7, *P* = 0.17).

**Table 2.** Comparison of 90-day medical complications between groups.

Medical Complications	Testosterone (*N* = 677)	Controls (*n* = 2554)	Odds Ratio	95% Confidence Interval	*P*-value
Myocardial Infarction	2 (0.3)	3 (0.1)	2.5	0.3–15	0.31
Pneumonia	8 (1.2)	35 (1.4)	0.9	0.4–1.8	0.7
Pulmonary embolism	0 (0.0)	12 (0.5)	NA	NA	NA
Deep vein thrombosis	0 (0.0)	9 (0.4)	NA	NA	NA
Urinary tract infection	13 (1.9)	33 (1.3)	1.5	0.8–2.8	0.22
Acute kidney injury	6 (0.9)	25 (1.0)	0.9	0.3–2.1	0.83
Blood transfusion	0 (0.0)	9 (0.4)	NA	NA	NA
Wound dehiscence	1 (0.1)	4 (0.2)	0.9	0.1–6.	0.96
Emergency department (ED) visits	57 (8.4)	180 (7.0)	1.2	0.9–1.6	0.22
Readmissions	13 (1.9)	74 (2.9)	0.7	0.4–1.2	0.17

*Reported as *n* (%).

### 5-Year surgical complications

At five years, testosterone users were found to have a significantly higher risk of PJI compared to controls (4.1% vs. 2.4%, OR 1.8, 95% CI 1.1–2.8, *P* = 0.015) as shown in Table 3. However, there were no significant differences in other long-term surgical complications, including all-cause revision (3.2% vs. 2.8%, OR 1.2, *P* = 0.55), dislocation (2.4% vs. 2.2%, OR 1.1, *P* = 0.84), aseptic loosening (4.1% vs. 5.6%, OR 1.00, *P* = 0.98), periprosthetic fracture (0.3% vs. 0.4%, OR 0.75, *P* = 0.72), incision and drainage (2.8% vs 2.0%, OR 1.5, *P* = 0.18), or stiffness (10.5% vs. 10.5%, OR 1.0, *P* = 0.99).

**Table 3.** Comparison of 5-year surgical complications between groups.

Surgical Complications	Testosterone (*N* = 677)	Controls (*n* = 2554)	Odds Ratio	95% Confidence Interval	*P*-value
All-cause revision	22 (3.2)	72 (2.8)	1.2	0.7–1.9	0.55
Periprosthetic joint infection	28 (4.1)	61 (2.4)	**1**.**8**	**1.1–2.8**	**0**.**015**
Dislocation	16 (2.4)	57 (2.2)	1.1	0.6–1.8	0.84
Aseptic loosening	28 (4.1)	144 (5.6)	1	0.7–1.4	0.98
Periprosthetic fracture	2 (0.3)	10 (0.4)	0.8	0.1–2.9	0.72
Incision and drainage	19 (2.8)	50 (2.0)	1.5	0.8–2.4	0.18
Stiffness	71 (10.5)	268 (10.5)	1	0.8–1.3	0.99

*Reported as *n* (%). Bold indicates statistical significance (*P* < 0.05).

## Discussion

This retrospective cohort study analyzed the effects of TRT on short- and medium-term postoperative outcomes in patients undergoing primary TSA. Notably, this analysis also used propensity-matching for age, sex, CCI score, obesity, tobacco use, and, importantly, a history of diagnosed hypogonadism to help distinguish the effects of TRT from those of such confounding variables. Compared to nonusers, there was no increased risk of medical complication in the 672 testosterone users within 90 days after surgery including, MI, pneumonia, PE, DVT, AKI, and wound dehiscence. At 5 years postoperatively, patients taking TRT prior to TSA demonstrated a higher risk of PJI (4.1% vs. 2.4%, OR 1.76), but there were no differences observed in revision rates, dislocation, aseptic loosening, periprosthetic fracture, or stiffness. These findings underscore the need for careful perioperative management of TSA patients on TRT and should encourage arthroplasty surgeons to consider engaging their endocrine colleagues in order to mitigate the risk of infection in these patients. The data also supported our hypothesis that there would be a statistically significant increase in the rates of PJI in the testosterone using cohort at 5 years; however, there were no differences in other periprosthetic complications compared to the matched controls.

Our findings reinforce the current theory that testosterone supplementation can increase infection rates after TSA,^9,27,29,30^ and expand upon it by investigating midterm implant-related outcomes using a large database with propensity matching and a narrower TRT inclusion criteria. The prior study by Su et al.^9^ found a 3.4% rate of PJI when TRT was used within 6 months of surgery compared to 2.4% in nonusers (OR 1.44, *P* = 0.042), similar to but less than our rate of 4.1%–2.4% (OR 1.76, *P* = 0.015). Notably, however, this finding was not statistically significant in the multivariate analysis of their study.^9^ This difference could be attributed to our method of matching using the CCI, which encompasses a broad range of comorbidities. The CCI in their investigation varied between the cohorts such that it was lower in the TRT group. As such, fewer pre-existing medical comorbidities existed in their TRT group which could have contributed to infection, thereby artificially lowering the PJI rate. Our analysis is also matched by age and gender and therefore controls for these confounders, which have been established as independent risk factors of PJI after primary TSA.^19,23–26^^,30,31^ Similarly, matching cohorts by other known contributors to potential surgical infection such as clinical obesity^31^ and tobacco use^32,33^ increases the reliability of these findings despite a smaller overall cohort.

Furthermore, by supporting the current literature connecting increased testosterone to PJI, this study advances ongoing discussions about why the association exists. Falconer et al.^25^ and Matsen et al.^29^ have both observed significantly increased rates of PJI in males while Schiffman et al.^27^ and Matsen et al.^30^ identified TRT and higher levels of serum testosterone as risk factors for increased skin loads of *Cutibacterium*, a commensal gram-positive rod abundant in the pilosebaceous units of the dermis and subdermis.^27^ These findings are notably important to the present study because the most commonly cultured organism in PJI of the shoulder is *C. acnes*.^23,27,29,30^ In fact, it has been documented that >70% of specimens cultured from unprepared skin surfaces in patients prior to primary TSA are positive for *C. acnes*.^27,30^ Moreover, increased preoperative load of topical *C. acnes* is known to be correlated with higher amounts in deep tissues and predictive of culture-positive PJI, further establishing this organism's infectious potential in primary TSA.^9,29,34,35^ Another small retrospective review by Matsen et al^29^ also helped confirm this. After evaluating the causes of PJI in 342 TSA revisions, they found that testosterone supplementation was a risk factor for the cases with a *C. acnes* source (8% vs. 2%, *P* = 0.02). While the database used in this investigation does not confirm infectious sources, it does provide a much larger case volume than previous studies and provides strong support for the connection between testosterone levels and a significantly increased risk of PJI in TSA. Although *C. acnes* is the organism most frequently linked to shoulder PJI, the PearlDiver database does not identify causative organisms. Therefore, we cannot confirm that PJI cases in TRT users were due to *C. acnes*, and the mechanistic interpretation should be viewed as hypothesis-supporting rather than confirmatory.

This study also offers additional information about crucial implant-related outcomes in these patients, demonstrating that despite a greater risk of infection, rates of aseptic loosening, dislocation, fracture, and stiffness remain the same. While two previous studies have published on a meaningful connection between TRT and PJI, both studies are underpowered such that the first did not find statistical significance in their final analysis and the second evaluated just a few hundred cases—half of our study size.^9,29^ Meanwhile, no studies have previously reported on these kinds of implant-related outcomes after TRT use. By doing so, this investigation offers novel information about the other sequelae of testosterone supplementation in the setting of primary anatomic TSA. Though infection has been frequently associated with postoperative complications such as component loosening, pain, and instability after shoulder arthroplasty,^23,36,37^ our findings suggest that TRT is not a major source of periprosthetic complications. The lack of difference in such complications between groups, namely loosening, dislocation, and periprosthetic fracture, could also be due to our controlling for tobacco use and hypogonadism using propensity matching, as they are known to independently affect bone quality negatively.^10,38,39^ This finding is particularly interesting because it concentrates the risks of testosterone supplementation on infection; however, it is still possible TRT could increase the rates of other periprosthetic complications not accounted for here.

With regard to our TRT inclusion criteria, our study's testosterone use group was comprised of patients who had used within 3 months of surgery versus 6 months in the analysis by Su et al.^9^ Three months remains consistent with prescribed TRT regimens and helps reduce the chances that a patient may stop or become inconsistent in their therapy.^39,41^ Ultimately, while TRT within 6 months of surgery was not found to be an independent risk factor in the investigation by Su et al.,^9^ these findings indicate that 3 months of TRT leading up to TSA confers a statistically significant increase in PJI risk. Since most TRT users continued therapy for years after TSA, it is unclear whether infection risk is driven primarily by perioperative exposure, postoperative continuation, or cumulative duration. An important distinction between our study and prior work by Su and colleagues is that 91% of patients in our TRT cohort continued therapy after surgery. This ongoing exposure likely contributes to the higher observed PJI OR (1.76 vs. 1.44). Prolonged postoperative testosterone exposure may sustain elevated sebum production and *C. acnes* burden beyond the immediate perioperative period, potentially increasing cumulative infection risk. Thus, the findings of this study may be plausibly explained by chronic TRT exposure. Future prospective work should evaluate whether temporary cessation before or after TSA mitigates infection risk.

While the average age of this matched analysis was 64 years, consistent with the average age of primary TSA patients reported in the literature,^6,9,16,17,31^ recent evidence has shown the use of TRT in this population has increased 7.5-fold in the last decade.^9^ TRT can be prescribed for a number of reasons including sexual dysfunction, mood disturbances, decreased muscle mass and bone mineral density, impaired cognition, and declines in subjective vitality.^11^ One of the most common reasons to prescribe therapy in men of this age, however, is hypogonadism.^9,11,39–41^ In addition to the previously mentioned factors, this study also matched cohorts by the presence of hypogonadism. As a result, our primary finding of increased rate of PJI with TRT isolates the association to the testosterone therapy itself instead of to the constellation of hormonal changes that may accompany diagnosed hypogonadism. However, this may obscure the relationship underlying how serum testosterone levels influence infection risk, since patients with hypogonadism on TRT could still have serum levels lower than a non-user without hypogonadism. Nevertheless, with the prevalence of TRT in patients undergoing TSA increasing,^8,9^ these data become critical in helping to better characterize the effects of exogenous testosterone supplementation on surgical outcomes.

This study is limited by its reliance on retrospective patient data, which is susceptible to coding inaccuracies that likely affect both cohorts similarly; thus, caution is warranted in interpreting significant associations as causative. The lack of granularity on the type, dose, administration route, serum testosterone level, and the absence of infectious organism identification also restrict subgroup analysis. TRT obtained outside insurance channels may not be captured, introducing potential misclassification. Therefore, the presence of organisms including *C. acnes* cannot be confirmed as the infectious agent, despite associations found by Matsen et al.^29^ and Schiffman et al.^27^ (*P* < 0.001). The use of billing codes limits access to functional and patient-reported outcomes and prevents differentiation among implant types, preoperative rotator cuff integrity, and other shoulder-specific metrics that could impact surgical decisions. As PearlDiver is a U.S.-based, non-randomized database, results may not generalize internationally. Nonetheless, the study's strengths include the PearlDiver database's large, nationally representative patient cohort and the use of propensity-matching, including for hypogonadism, which mitigates potential biases and enhances the reliability of findings on TSA complication profiles in TRT patients, offering clinical insights. While the cohort size was adequate to evaluate the primary endpoint of PJI, several secondary outcomes were infrequent and associated with wide CIs, suggesting potential underpowering for rare complications such as periprosthetic fracture. Because this was a retrospective analysis of all eligible patients within a fixed administrative database, an a priori power calculation was not performed. Accordingly, non-significant findings for low-incidence outcomes should be interpreted cautiously, as the absence of statistical significance does not exclude clinically meaningful differences. Future studies should investigate the optimal timing for stopping TRT prior to shoulder arthroplasty.

## Conclusions

Perioperative testosterone use was associated with a significantly higher risk of PJI following primary TSA, but no differences were found in rates of other major complications including revision rates, dislocation, aseptic loosening, periprosthetic fracture, and stiffness. These findings support the need for preoperative identification of TRT use and coordinated discussion with endocrinology regarding optimal perioperative management. The appropriate timing of TRT cessation and resumption remains unknown and warrants future study.

## Supplemental Material

sj-docx-1-sel-10.1177_17585732261437972 - Supplemental material for Perioperative testosterone therapy linked to higher 5-year risk of periprosthetic joint infection without increased 90-day major complications or revision rates in total shoulder arthroplasty Supplemental material, sj-docx-1-sel-10.1177_17585732261437972 for Perioperative testosterone therapy linked to higher 5-year risk of periprosthetic joint infection without increased 90-day major complications or revision rates in total shoulder arthroplasty by Jad J Lawand, Connor Crutchfield, Alejandro M Holle, Olawale A Sogbein, Sameer R Khawaja, Umar Ghilzai, Brian W Hill, Adam Z Khan, John G Horneff and Joseph A Abboud in Shoulder & Elbow
