# Supplementary material for: Perioperative testosterone therapy linked to higher 5-year risk of periprosthetic joint infection without increased 90-day major complications or revision rates in total shoulder arthroplasty
Source: Shoulder Elbow. 2026 Apr 7:17585732261437972. Online ahead of print. doi: 10.1177/17585732261437972 (PMC13056804; doi:10.1177/17585732261437972)
Supplement: sj-docx-1-sel-10.1177_17585732261437972 - Supplemental material for Perioperative testosterone therapy linked to higher 5-year risk of periprosthetic joint infection without increased 90-day major complications or revision rates in total shoulder arthroplasty [file sj-docx-1-sel-10.1177_17585732261437972.docx]

Supplementary Table 1: CPT and ICD Codes Used

| Primary TSA | CPT-23472, ICD-9-P-8188, ICD-10-P-0RRJ00Z, ICD-10-P-0RRK00Z, ICD-9-P-8180, ICD-10-P-0RRJ0JZ, ICD-10-P-0RRK0JZ, ICD-10-P-0RRJ07Z, ICD-10-P-0RRK07Z, ICD-10-P-0RRK0KZ, ICD-10-P-0RRJ0KZ, ICD-10-P-0RRJ0J6, ICD-10-P-0RRK0J6, ICD-10-P-0RRJ0J7, ICD-10-P-0RRK0J7 |
| --- | --- |
| Testosterone Replacement Therapy | RUG-4-DIHYDROTESTOSTERONE, DRUG-DEPO-TESTOSTERONE, DRUG-ESTROGEN-METHYLTESTOSTERONE, DRUG-ESTROGEN_&_METHYLTESTOSTERONE, DRUG-FIRST-TESTOSTERONE, DRUG-FIRST-TESTOSTERONE_MC, DRUG-METHYLTESTOSTERONE, DRUG-METHYLTESTOSTERONE_MICRONIZED, DRUG-TESTOSTERONE, DRUG-TESTOSTERONE_CYPIONATE, DRUG-TESTOSTERONE_CYPIONATE_MICRO, DRUG-TESTOSTERONE_ENANTHATE, DRUG-TESTOSTERONE_MICRONIZED, DRUG-TESTOSTERONE_PROPIONATE |
| **Exclusion** | |
| Fracture | ICD-9-D-73311, ICD-9-D-81200, ICD-9-D-81201, ICD-9-D-81202, ICD-9-D-81203, ICD-9-D-81209, ICD-9-D-81210, ICD-9-D-81211, ICD-9-D-81212, ICD-9-D-81213, ICD-9-D-81219, ICD-9-D-81220, ICD-9-D-81221, ICD-9-D-81230, ICD-9-D-81231, ICD-9-D-81240, ICD-10-D-M84429A, ICD-10-D-M84421A, ICD-10-D-M84421D, ICD-10-D-M84421G, ICD-10-D-M84421K - ICD-10-D-M84629S) , ICD-10-D-S42201A - ICD-10-D-S42409S |
| Malignancy | ICD-9-D-1703, ICD-10-D-C413, ICD-9-D-1704, ICD-10-D-C4000, ICD-9-D-1705, ICD-10-D-C4010, ICD-9-D-1709, ICD-10-D-C419, ICD-9-D-1712, ICD-10-D-C4910, ICD-9-D-1954, ICD-10-D-C7640, ICD-9-D-1963, ICD-10-D-C773 |
| **Surgical Complications** | |
| Revision | CPT-23473, CPT-23474, ICD-9-P-8197, ICD-10-P-0RWJ0JZ, ICD-10-P-0RWJ0KZ, ICD-10-P-0RWK0JZ, ICD-10-P-0RWK0KZ, ICD-10-P-0RWJ37Z, ICD-10-P-0RWJ3JZ, ICD-10-P-0RWK37Z, ICD-10-P-0RWK3JZ, ICD-10-P-0RWJ3KZ, ICD-10-P-0RWK3KZ, ICD-10-P-0RWJ47Z, ICD-10-P-0RWJ4JZ, ICD-10-P-0RWJ4KZ, ICD-10-P-0RWK47Z, ICD-10-P-0RWK4JZ, ICD-10-P-0RWK4KZ, ICD-10-P-0RWJX8, ICD-10-P-0RWJXJ, ICD-10-P-0RWJXK, ICD-10-P-0RWKX8, ICD-10-P-0RWKXJ, ICD-10-P-0RWKXK |
| Periprosthetic Fracture | ICD-9-D-99644, ICD-10-D-M9731XA, ICD-10-D-M9732XA, ICD-10-D-M978XXA, ICD-10-D-M979XXA |
| Prosthetic Joint Infection | ICD-10-D-T8450XA, ICD-10-D-T8450XD, ICD-10-D-T8450XS, ICD-10-D-T8459XA, ICD-10-D-T8459XD, ICD-10-D-T8459XS, ICD-9-D-99666 |
| Aseptic Loosening | ICD-9-D-99647, ICD-10-D-T84098A, ICD-9-D-99641, ICD-9-D-99677, ICD-10-D-T84038A, ICD-9-D-99649, ICD-10-D-T84498A, ICD-10-D-T8489XA |
| Stiffness | ICD-9-D-71951, ICD-10-D-M25611, ICD-10-D-M25612, ICD-10-D-M25619 |
| Dislocation | ICD-9-D-99642, ICD-10-D-T84028A, ICD-10-D-T84029A |
| Incision and Drainage | CPT-29820, CPT-29821, CPT-29822, CPT-2982 |
